# The different faces of metastatic melanoma in the gastrointestinal tract

**DOI:** 10.1186/s13244-022-01294-5

**Published:** 2022-10-04

**Authors:** Eva Mendes Serrao, Ana Maria Costa, Sergio Ferreira, Victoria McMorran, Emma Cargill, Caroline Hough, Ashley S. Shaw, Brent O’Carrigan, Christine A. Parkinson, Pippa G. Corrie, Timothy J. Sadler

**Affiliations:** 1 Department of Radiology, Addenbrooke’s Hospital, Cambridge University Hospitals NHS Foundation Trust, Cambridge, UK; 2 Department of Radiology, University of Cambridge, Box 218, Cambridge, CB2 0QQ UK; 3 Department of Radiology, Hospital Fernando Fonseca, Amadora, Portugal; 4 Department of Oncology, Addenbrooke’s Hospital, Cambridge University Hospitals NHS Foundation Trust, Cambridge, UK

**Keywords:** Melanoma, Metastases, Gastrointestinal tract, Tomography (X-ray computed), Magnetic resonance imaging

## Abstract

Melanoma is the most aggressive form of skin cancer, with tendency to spread to any organ of the human body, including the gastrointestinal tract (GIT). The diagnosis of metastases to the GIT can be difficult, as they may be clinically silent for somewhile and may occur years after the initial melanoma diagnosis. CT imaging remains the standard modality for staging and surveillance of melanoma patients, and in most cases, it will be the first imaging modality to identify GIT lesions. However, interpretation of CT studies in patients with melanoma can be challenging as lesions may be subtle and random in distribution, as well as sometimes mimicking other conditions. Even so, early diagnosis of GIT metastases is critical to avoid emergency hospitalisations, whilst surgical intervention can be curative in some cases. In this review, we illustrate the various imaging presentations of melanoma metastases within the GIT, discuss the clinical aspects and offer advice on investigation and management. We offer tips intended to aid radiologists in their diagnostic skills and interpretation of melanoma imaging scans.

## Key points

- Melanoma is the most common solid tumour metastasising to the GIT.
- Melanoma metastases in the GIT can have multiple radiological appearances and mimic other conditions.
- Radiological identification of melanoma metastases in the GIT is important, as early diagnosis and treatment improve quality and quantity of life, even in palliative cases.

## Background

Melanoma is the most aggressive form of skin cancer. The major risk factors for cutaneous melanoma include exposure to ultraviolet rays, fair complexion and prior personal or family history of melanoma. Disease outcomes depend on the extent and stage of melanoma at presentation. Whilst there has been a steady increase in the incidence of melanoma over the past decades [1], the mortality rate has decreased for all stages of disease [2]. This is thought to be mostly due to earlier diagnosis, surgical intervention and active systemic therapies [3]. In particular, the 5-year survival rate of metastatic melanoma has increased from less than 5% in 2010 to now around 30% [4].

Melanoma invades locally and spreads via lymph nodes and the bloodstream to distant organs. Common sites of metastasis include the liver, lungs, skin and brain [5]. The gastrointestinal tract (GIT) is a less common site for melanoma metastases to occur in [6]. Cutaneous melanoma is the most common tumour metastasising to the GIT [7, 8]; however, its diagnosis remains challenging due to its vague clinical presentation and diverse morphological appearance on imaging. Computed tomography (CT) is the standard modality for detection, staging and surveillance of patients with melanoma. Functional imaging, such as positron emission tomography (PET)-CT and whole body multiparametric magnetic resonance imaging (MRI), is utilised in specific circumstances [9]. Depending upon which guidelines are followed, PET-CT can be an alternative imaging test for staging and surveillance [9, 10]. As for whole body MRI, current guidelines only recommend its use in staging and surveillance of pregnant and young (< 24 years old) patients [9].

**Fig. 1:**
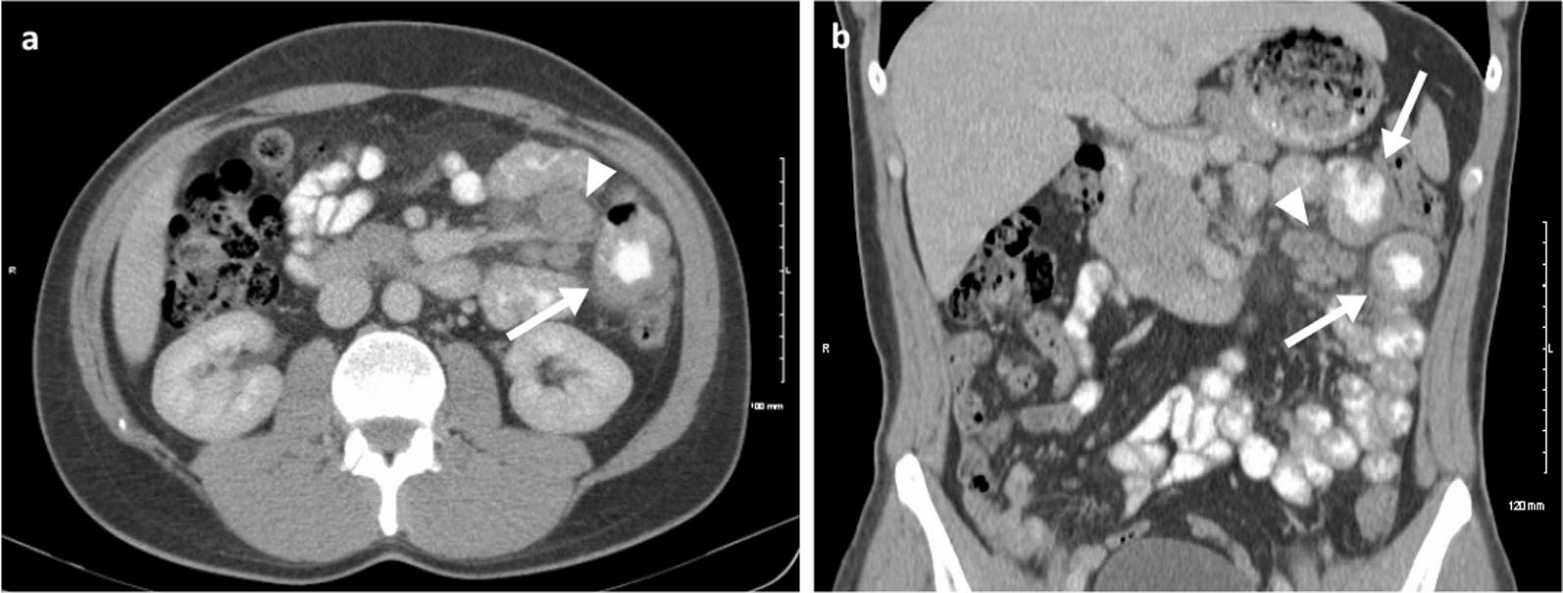
Case report: oral and intravenous portal phase contrast-enhanced CT axial (**a**) and coronal (**b**) of a 33-year-old male patient diagnosed with left lower eyelid melanoma which subsequently metastasised to the lymph nodes, lungs, brain, SB and subcutaneous tissue in the following 5-year period. Complete response was achieved after chemotherapy, immunotherapy and radiotherapy. One year after treatment completion he relapsed, presenting with two jejunal metastases (arrows) and involvement of the draining mesenteric lymph nodes (arrow heads). Surgical resection was then performed with no evidence of disease recurrence to date, after 10 years of follow-up

Despite GIT metastases suggesting overall poorer prognosis and survival [6], some patients achieve long-term remission following surgical resection (in example Fig. 1) [11]. Although much of the survival gains of patients treated for metastatic melanoma (MM) are nowadays attributable to modern systemic therapies, surgical clearance of oligometastatic disease (typically defined as up to 3 disease sites) remains a key intervention for alleviation of symptoms and improving survival, even in patients responding to systemic therapy [6, 12, 13]. Therefore, early diagnosis and rapid intervention for melanoma in the GIT are important to maximise both quantity and quality of life [14].

Melanoma in the GIT can also rarely be a true primary tumour arising from the GI mucosa, with this entity being biologically distinct from cutaneous melanoma. In the GIT, they arise most frequently in the anorectal mucosal epithelium (anus 31% and rectum 22%), and less often in the oesophagus (6%), stomach (3%), small intestine (2%) and large intestine (1%) [15], with a high proportion arising in the mucosal linings of the oral-nasopharynx (35%).

Evaluation of staging CT studies in patients with melanoma can be difficult and time consuming given the extent and unpredictable pattern of disease. Nevertheless, the GIT is an important review area in these patients, particularly in the case of primary cutaneous melanoma arising from the head and neck region, trunk and lower extremity [16] as these are the primary melanoma sites that more commonly metastasise to the GIT. In this review, we aim to illustrate the various imaging features of melanoma metastases in the GIT with some tips and a brief discussion on the clinical aspects. This knowledge will hopefully aid radiologists in their interpretation of scans undertaken in melanoma patients.

**Tip 1**: Melanoma is the most common cancer type that metastasises to the GIT.

**Tip 2**: Radiological detection and reporting of melanoma metastases in the GIT are important, as early diagnosis and treatment improve quality and quantity of life.

### Oesophagus and stomach

**Fig. 2:**
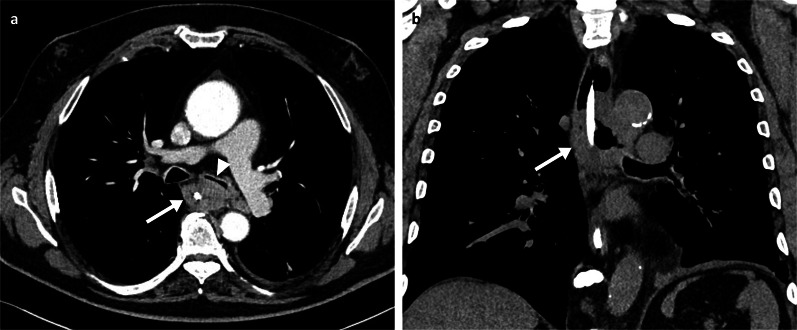
Arterial phase contrast-enhanced CT axial (**a**) and non-enhanced coronal (**b**) of a 58-year-old male patient demonstrating a large oesophageal metastatic melanoma mass (arrows) with luminal narrowing and compression of the left main bronchus (arrowhead). A nasogastric tube is in situ

Primary melanoma of the oesophagus is rare, representing less than 0.1–0.5% of all oesophageal malignant tumours [17]. Since first described in 1895 by Spielberg [18], metastatic involvement of melanoma in the oesophagus was shown to be even rarer than primary oesophageal melanoma, with a reported incidence of 4% in a series of 125 autopsy cases of cutaneous melanoma [19]. Symptoms are similar to those caused by other oesophageal tumours and include dysphagia, weight loss, haematemesis and/or melaena. Metastatic oesophageal lesions are either mucosal or submucosal [19], with both contrast studies and endoscopy providing a reasonable diagnostic yield. Contrast barium oesophagram often reveals a polypoid intraluminal filling defect with or without ulceration. CT scan of the chest may reveal the tumour, as an eccentric or circumferential wall thickening (Fig. 2) or as lesions protruding into the lumen. However, endoscopy with tissue sampling often provides the definitive diagnosis. Nevertheless, differentiation between primary melanoma or secondary MM is often difficult at the preoperative stage. Oesophageal melanosis is important as it is assumed to be a predisposing factor. However, this is only seen in one quarter of cases of oesophageal melanomas [17].

**Fig. 3:**
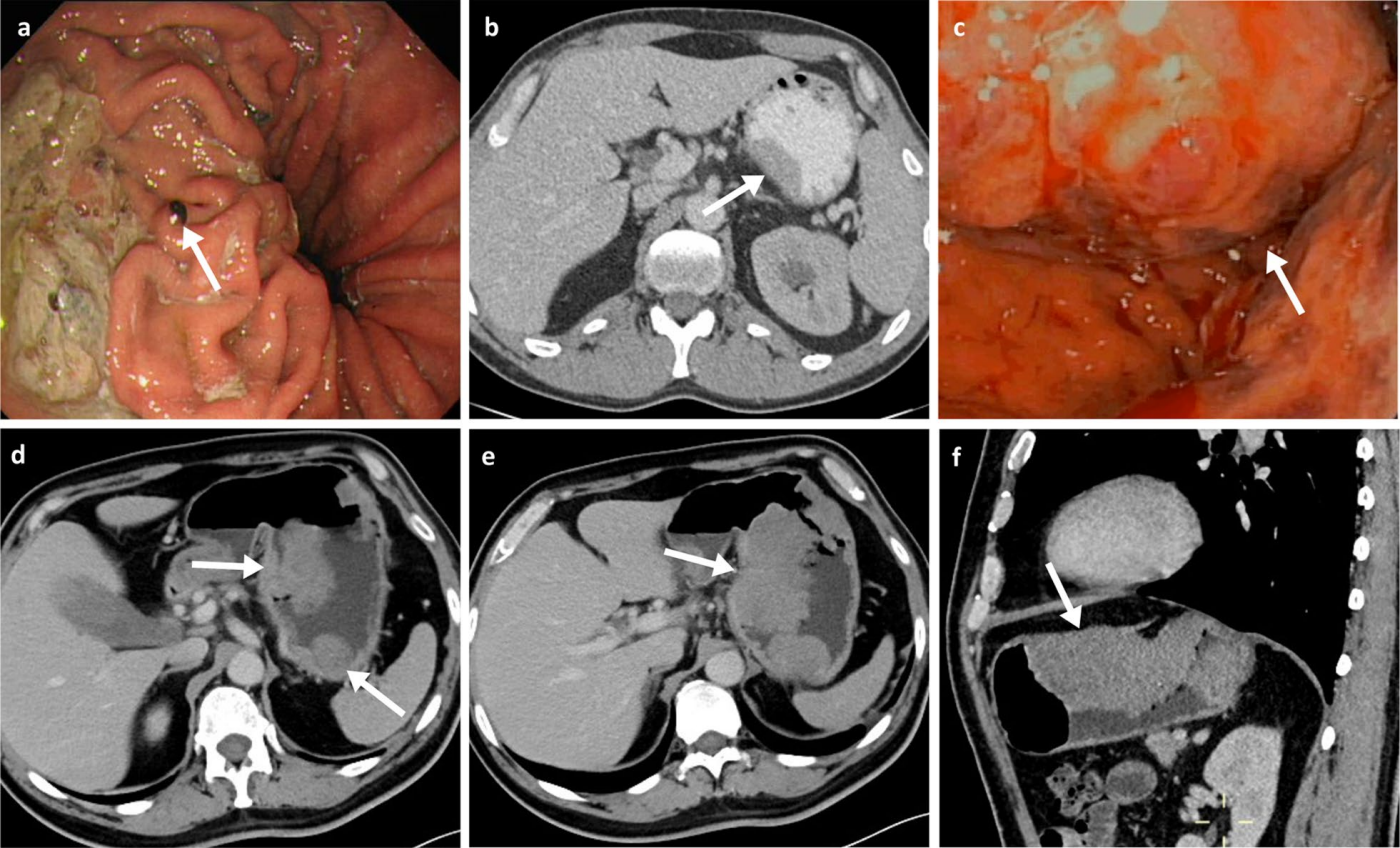
Gastric melanoma metastases. **a** Endoscopy of a 69-year-old female patient with a melanotic nodule in the proximal gastric body (arrow). **b** Oral and intravenous portal phase contrast-enhanced CT axial of a 42-year-old male patient with a sessile soft-tissue melanoma metastasis associated with the lesser curvature (arrow). **c**–**f** Endoscopy and portal phase contrast-enhanced CT axial and sagittal of a 51-year-old male patient demonstrating a large melanoma metastasis within the body of the stomach with resulting luminal narrowing (arrows)

The stomach, after the small bowel and colon, is the third most common GIT site involved by MM. Patients with MM in the stomach can present with nausea, vomiting, gastrointestinal bleeding, weight loss and occasionally with acute perforation. CT imaging can suggest the diagnosis by the presence of a mural nodule or mass (Fig. 3), with or without cavitation, but definitive diagnosis is best achieved by endoscopy and biopsy. However, there is growing evidence that MRI with diffusion weighted imaging (DWI) can provide improved early detection and characterisation of gastric lesions as well as local staging. The high cellularity and melanin content of melanoma metastases confer them with a high DWI signal, low ADC and in some cases a characteristic high T1 intensity [20–22].

Endoscopic classification of gastric metastases include: (a) melanotic nodules (Fig. 3a), often ulcerated at the centre; and also the most frequent endoscopic feature, (b) submucosal tumour, melanotic or not, elevated and ulcerated at the apex; providing the typical aspect of “bull’s eye” lesions in barium meals, and (c) mass lesions with varying incidence of necrosis and melanosis [23]. Sometimes, it may also appear as simple ulcers [24]. Most gastric metastases from MM occur at the greater curvature of the body and fundus, with the lesser curvature lesions being uncommon (Fig. 3) [25].

Metastatic melanoma involving the oesophagus and/or stomach at the time of diagnosis is considered a sign of disseminated disease and thus has a poor prognosis. Although surgical treatment has been attempted in some melanoma patients with oesophageal and gastric metastases, surgery seems to be of limited practical value and should be performed only in carefully selected patients or in patients with complications [26].

**Tip 3:** Review of the oesophagus and stomach should always be performed when interpreting CT scans of melanoma patients, as lesions can sometimes be asymptomatic.

**Tip 4:** When reviewing the oesophagus, look for eccentric or circumferential wall thickening and/or lesions protruding into the lumen.

**Tip 5:** In the stomach, look for any mass lesions along the greater curvature of the body and fundus, as melanoma metastases tend to occur particularly at these sites.

### Duodenum and small bowel

The small bowel is the most common metastatic site for melanoma in the GIT [19, 27]. Melanoma is the most common solid cancer type to metastasise to the small bowel (SB) [7, 8] with the jejunum and terminal ileum being the most commonly involved segments [28–30]. Although SB metastases are estimated to occur in up to 60% of patients with MM in post-mortem studies [19, 31, 32], clinical antemortem detection can be as low as 1–5% of cases [19]. Small bowel metastases are generally discovered either at the time of diagnosis, or not uncommonly several years after the primary malignancy (an average of around 7 years) [8]. This is believed to be due to the high expression of the chemokine ligand CCR9 in the small bowel, therefore promoting transmigration and homing of melanoma tumour cells which are known to have significant surface expression of the chemokine receptor CCR9 [33, 34]. Primary melanoma of the small bowel is rare, remaining a controversial diagnosis as it could be a metastasis from either an unidentified or a regressed primary cutaneous melanoma [35, 36].

The clinical presentation is usually non-specific, including vague abdominal pain, unexplained weight loss, iron-deficiency anaemia, change in bowel habits and GI bleeding, or even painless jaundice when in the duodenum [37], though a large portion will be asymptomatic. Rarely, patients can present with an acute abdomen due to intestinal obstruction, intussusception, perforation or fistula [27, 38–40]. As in other segments of the GIT, the diagnosis can be made by CT scanning, however endoscopy in the case of the duodenum and video-capsule for non-stenotic SB lesions are still the preferred methods to confirm the diagnosis [13]. CT/MR enterography/enteroclysis can be helpful for the non-invasive detection of small SB lesions. PET/CT is another option given its improved diagnostic accuracy over CT, with a sensitivity of 86% and specificity of 97% [27], and additional potential for the detection of other secondary lesions, unknown primary or residual tumour [12, 27, 41, 42]. Nonetheless, definitive diagnosis can only be obtained histologically through surgical/endoscopic biopsy.

**Fig. 4:**
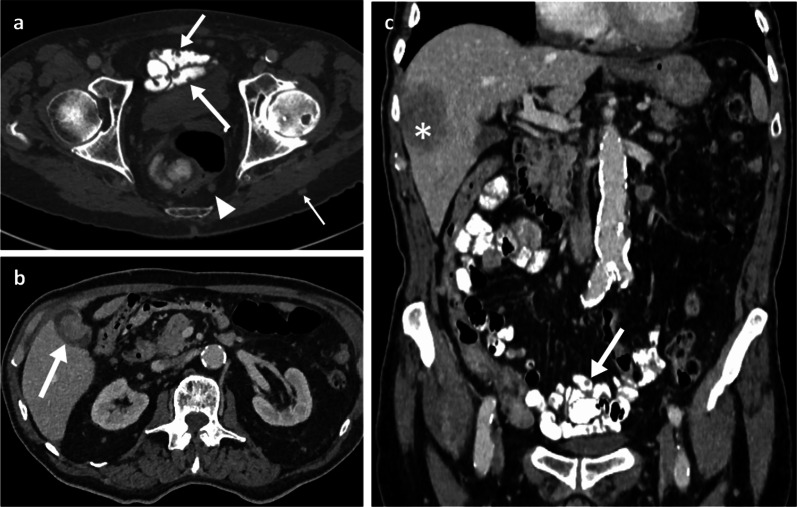
Oral and intravenous portal phase contrast-enhanced CT axial (**a**, **b**) and coronal (**c**) of an 85-year-old male patient with numerous small melanoma metastases throughout the bowel with nodular mucosal thickening (arrows in **a**, **c**), metastatic peritoneal nodules (arrowhead in **a**), gallbladder metastasis (arrow in **b**), liver metastasis (* in **c**) and subcutaneous metastasis (thin arrow in **a**)

**Fig. 5:**
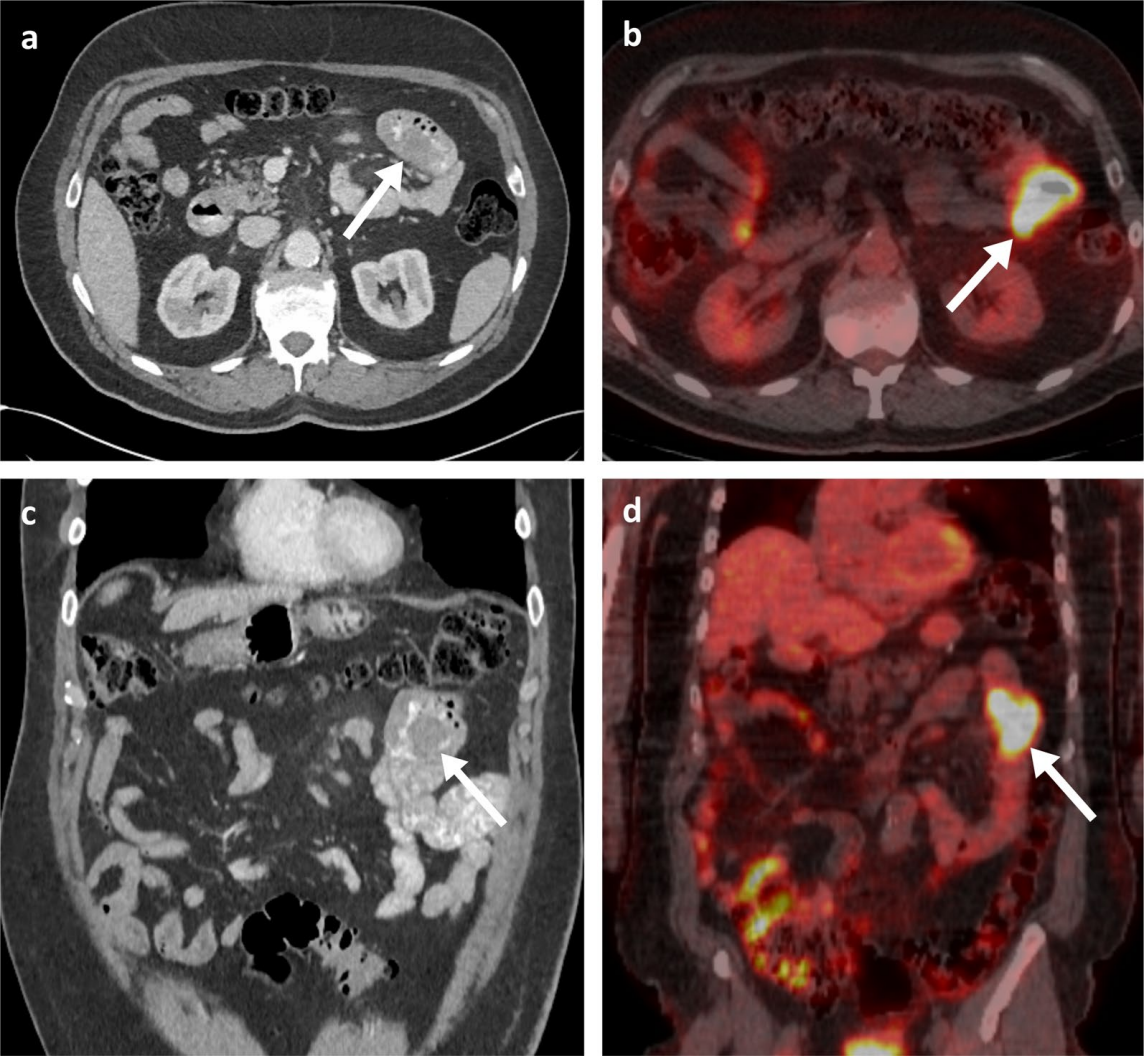
Oral and intravenous portal phase contrast-enhanced CT axial (**a**) and coronal (**c**) of a 70-year-old male patient with an eccentric mural melanoma metastasis within the proximal jejunum (arrows). PET-CT axial (**b**) and coronal (**d**) demonstrating FDG avidity with an SUVmax of 21.7 (arrows)

**Fig. 6:**
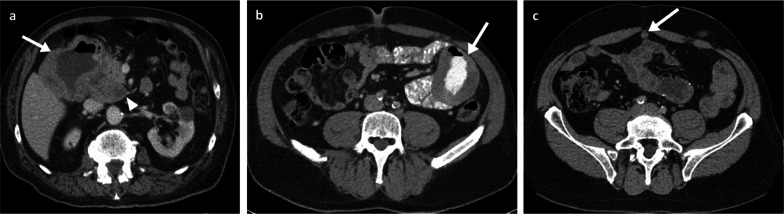
Duodenal and small bowel metastases. Portal phase contrast-enhanced CT axial (**a**) of an 80-year-old male patient with a large aneurysmal metastatic melanoma mass in the duodenum (arrow) and adjacent necrotic node (arrowhead). Oral and intravenous portal phase contrast-enhanced CT axial of a 57-year-old male patient with large jejunal melanoma metastasis (**b**: arrow) and anterior peritoneal nodule (**c**: arrow)

**Fig. 7:**
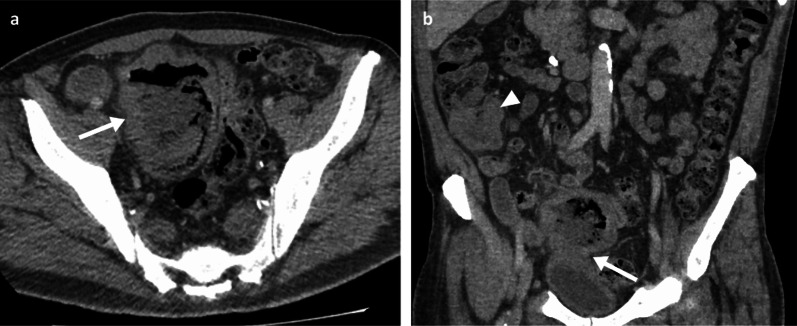
Portal phase contrast-enhanced CT axial and coronal (**a**, **b**) of a 71-year-old male patient demonstrating a large cavitating small bowel melanoma metastasis with urinary bladder tethering and dome invasion (arrows). Further separate caecal pole metastasis (arrowhead)

**Fig. 8:**
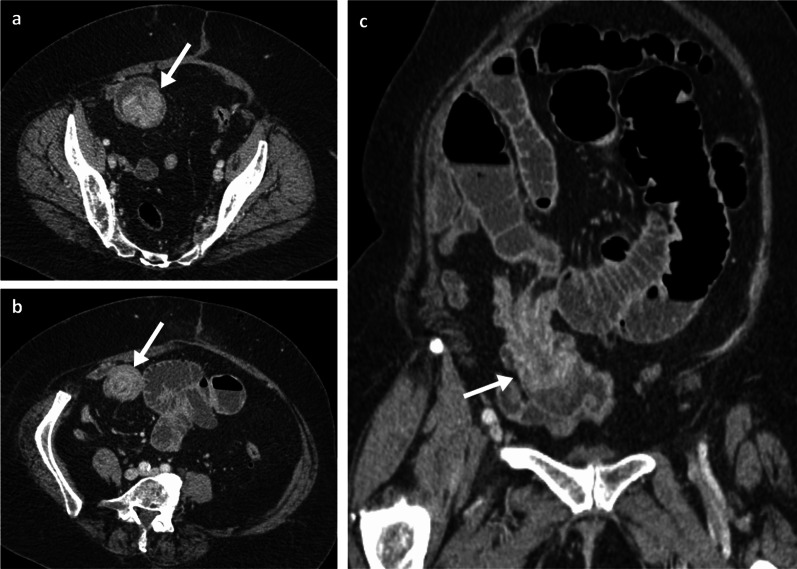
Portal phase contrast-enhanced CT axial (**a**, **b**) and oblique coronal (**c**) of a 71-year-old female patient with ileal melanoma metastasis with resulting obstructing intussusception (arrows)

As imaging patterns of duodenal/SB involvement can be diverse and mimic several other conditions, including other primary tumours, lymphoma or benign conditions such as infection or haemorrhage, it is important to keep melanoma as a possible differential in patients with a prior history of melanoma and/or with evidence of widespread disease involving other organs. In the duodenum and SB, the melanoma deposits more frequently present as polypoid nodules [43] (Fig. 4), and less frequently as ulcerating mural nodules/masses (Fig. 5), aneurysmal lesions which are classically attributed to lymphoma [44, 45] (Fig. 6), or infiltrating masses (Fig. 7). The classic finding on barium follow-through imaging is a target lesion or “bull's eye” lesion, but this is infrequently found. Additional detection of other ancillary features such as cystic lymphadenopathy (Fig. 6) and extra-intestinal metastatic lesions in unusual sites like soft tissues/subcutaneous fat, peritoneum and gallbladder (Figs. 4, 6), can point towards the correct diagnosis. Indeed, more than 50% of the patients with MM and GIT involvement will also have other sites and organs affected [6]. Also, the presence of SB intussusception in adults should raise concerns for metastatic melanoma (Fig. 8).

Despite the significant clinical impact of checkpoint inhibitors in patients with metastatic melanoma, operative management remains the mainstay treatment for small bowel melanoma, with growing evidence that early diagnosis and treatment can improve rates of survival [14], and quality of life even in palliative cases.

**Tip 6:** Melanoma is the most common primary malignancy to metastasise to the SB. Always consider melanoma in your differentials, particularly if there is a past medical history of melanoma.

**Tip 7:** Metastatic melanoma of the SB can have diverse appearances and mimic other diseases. Looking for extra-intestinal metastases is important to increase diagnostic confidence.

**Tip 8:** Be suspicious of metastatic melanoma in adult patients with SB intussusceptions, particularly if there is a past history of melanoma.

### Colon, rectum and anus

Melanoma metastases are uncommon in the large bowel, rectum and anus, with a predicted incidence of 15–22% in the colon [19, 27], 5% in the rectum and 1% in the anus [6, 19]. As in other parts of the GIT, primary mucosal melanomas can occur, with most arising in the anorectal lining. However, differentiation between primary melanoma and metastatic melanoma can still be difficult, often requiring careful histological assessment [39].

**Fig. 9:**
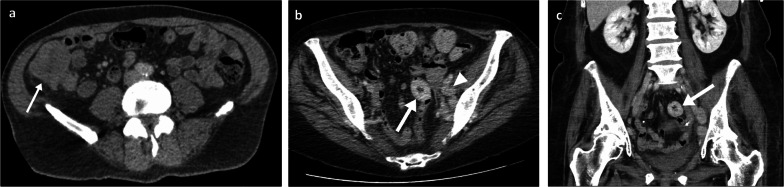
Colonic melanoma metastases. Portal phase contrast-enhanced CT axial (**a**) of a 71-year-old male patient with a caecal pole melanoma metastasis (arrow). Portal phase contrast-enhanced CT axial (**b**) and coronal (**c**) of 71-year-old female patient with a melanoma metastatic deposit to the sigmoid colon with circumferential thickening and enhancement (arrows) and pathological left iliac nodes (arrowhead)

**Fig. 10:**
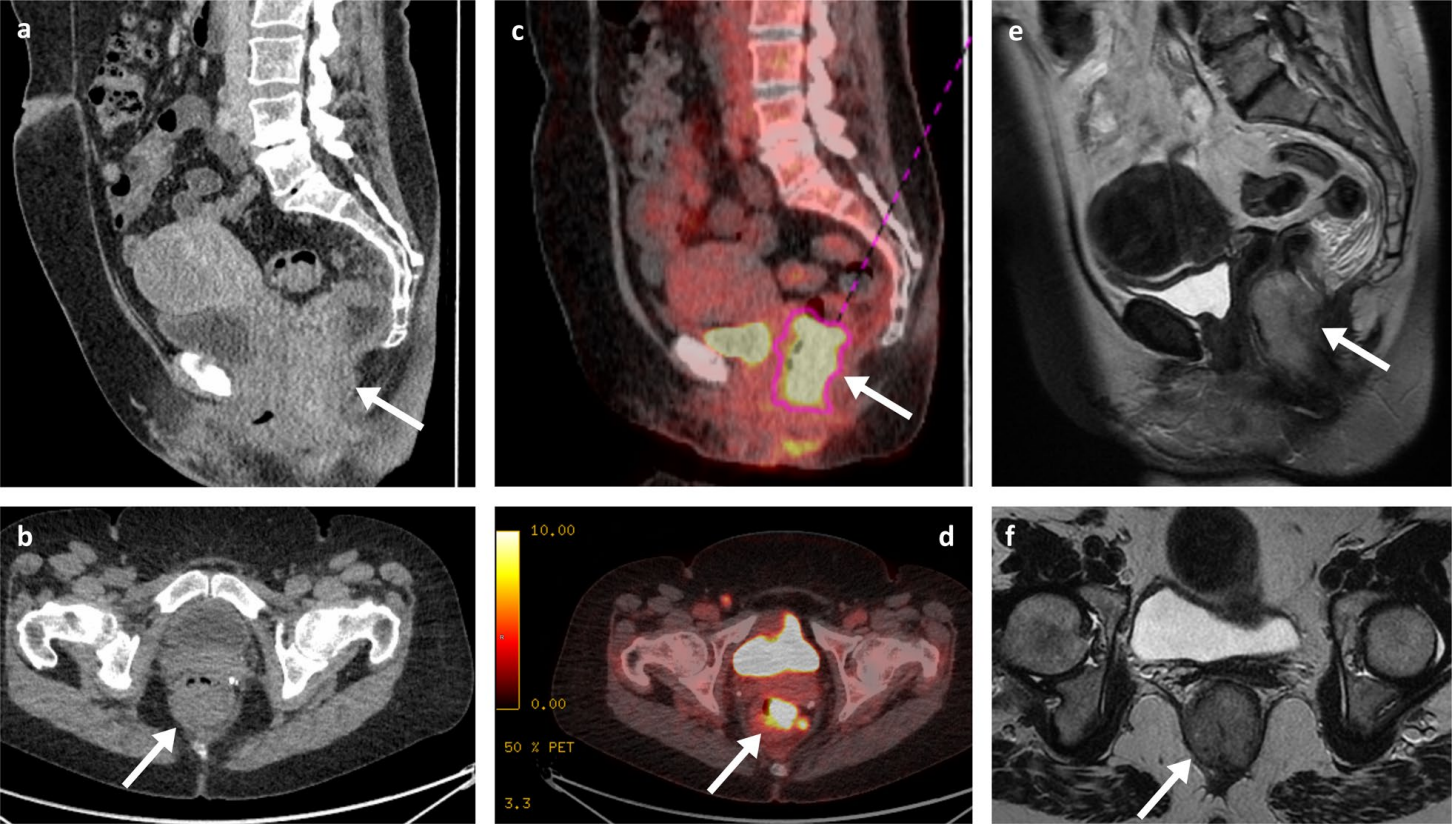
Portal phase contrast-enhanced CT sagittal (**a**) and axial (**b**), PET-CT sagittal (**c**) and axial (**d**), and MRI T2WI sagittal (**e**) and T2WI axial (**f**) of a 57-year-old female patient with an anorectal metastatic melanoma (arrows) with a craniocaudal length of 5 cm, situated 1.3 cm from the anal verge with involvement of the right internal sphincter complex

**Fig. 11:**
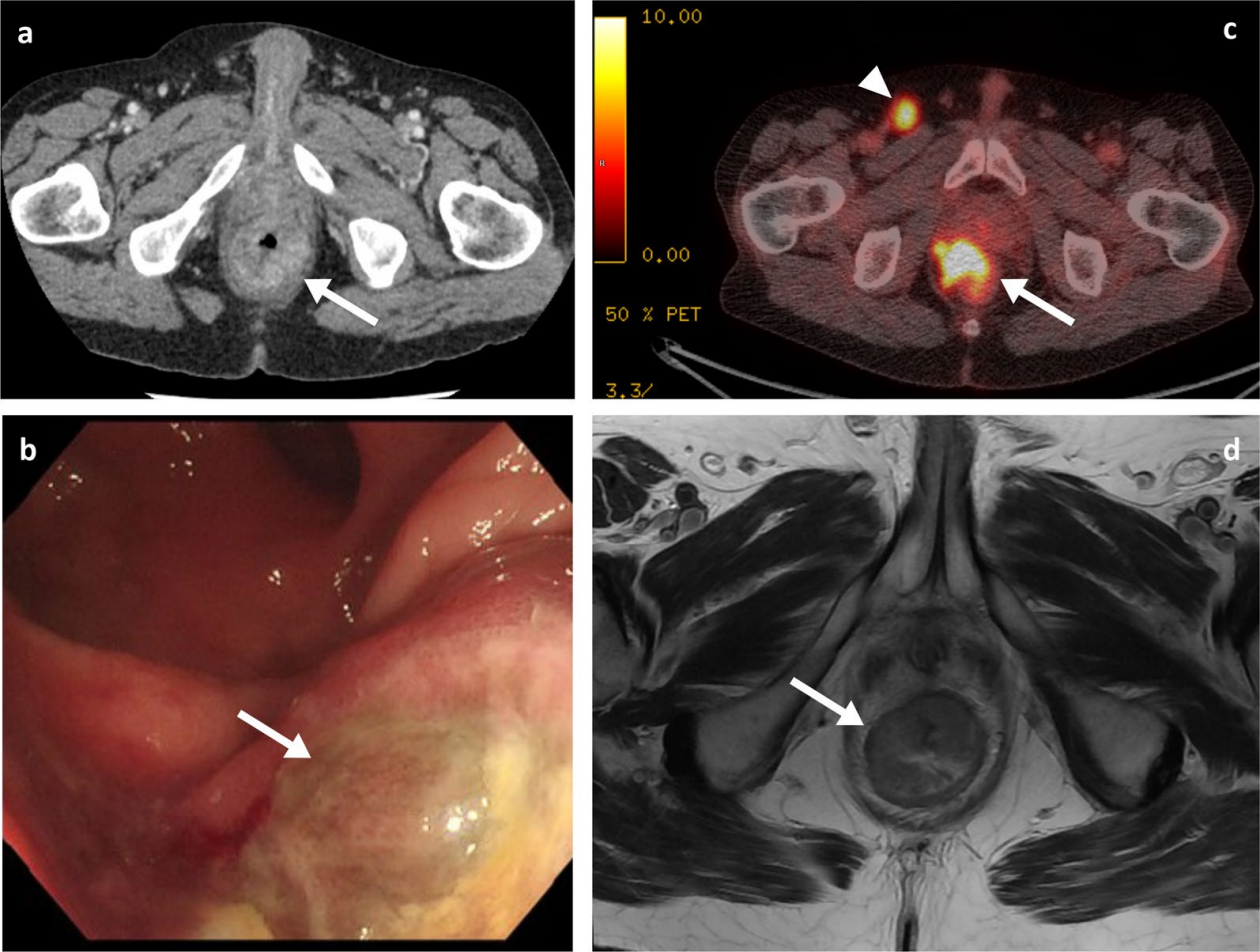
Portal phase contrast-enhanced CT axial (**a**), colonoscopy (**b**), PET-CT axial (**c**) and MRI T2WI axial (**d**) of a 64-year-old male patient with an infiltrative metastatic anorectal melanoma mass. The PET-CT (**c**) also demonstrates a pathological right inguinal node (arrowhead)

**Fig. 12:**
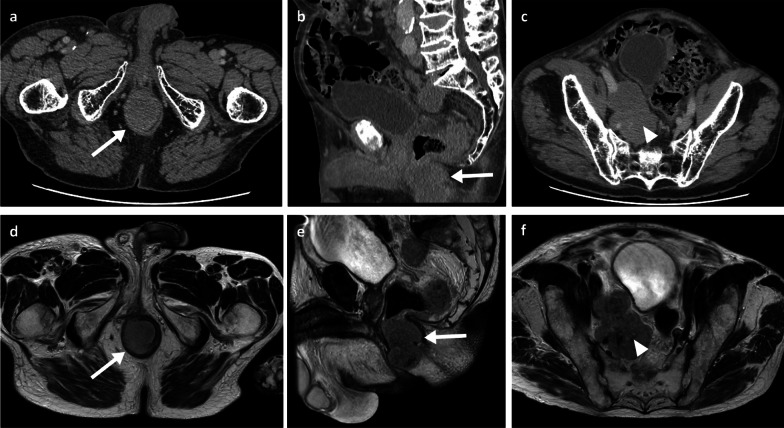
Portal phase contrast-enhanced CT axial (**a**, **c**) and sagittal (**b**), MRI T2WI axial (**d**, **f**) and T2WI sagittal (**e**) of a 77-year-old male patient with a metastatic melanoma anal canal mass (arrows) with extensive lymphadenopathy involving the mesorectal fat, iliac, inguinal and retroperitoneal chains (arrowheads)

**Fig. 13:**
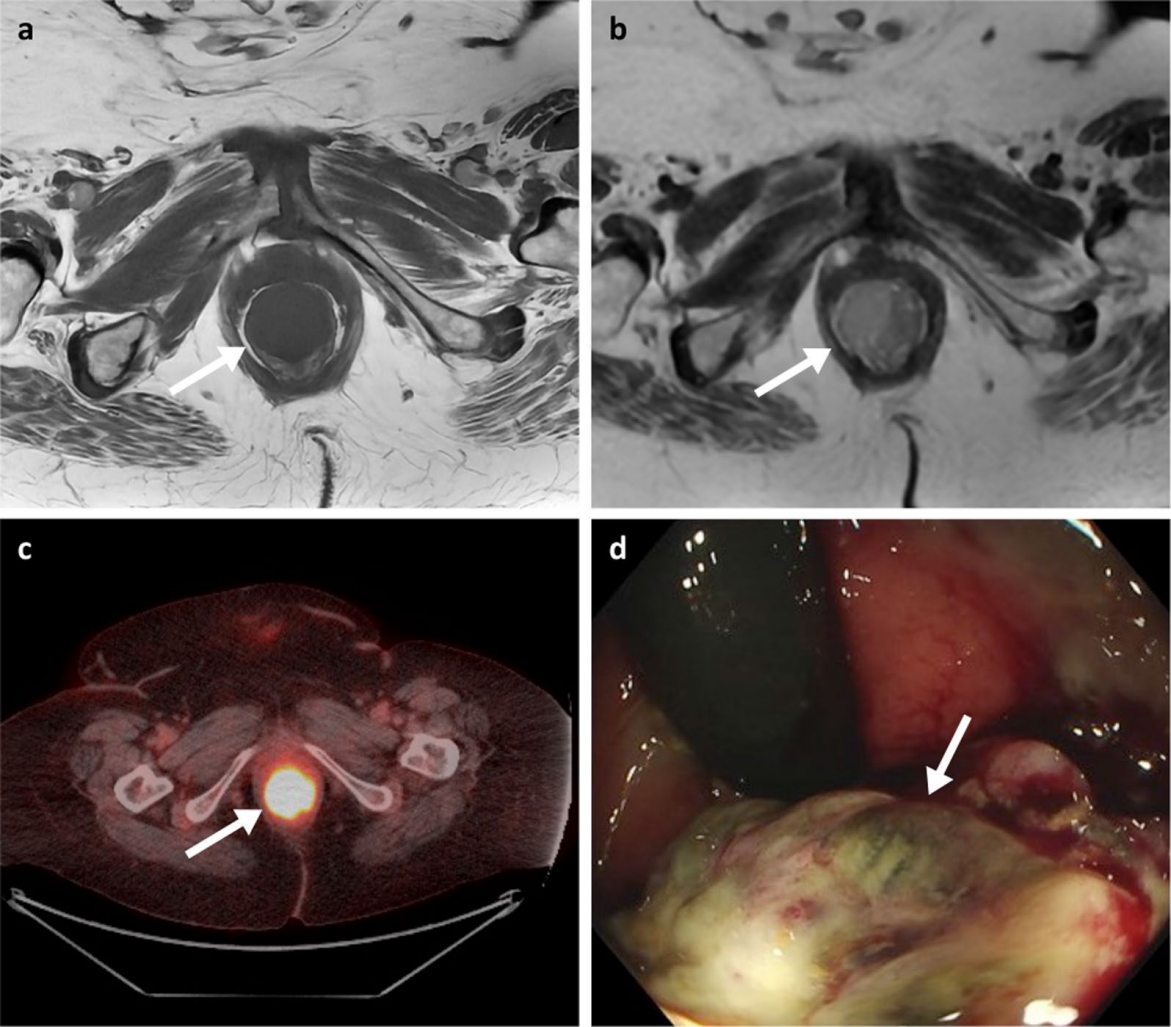
MRI T1WI and T2WI axial (**a**, **b**), PET-CT axial (**c**) and flexible sigmoidoscopy (**d**) of a 71-year-old female patient with a metastatic melanoma anorectal mass (arrows). Black pigment is visible within the ulcerated part of the lesion on sigmoidoscopy

**Fig. 14:**
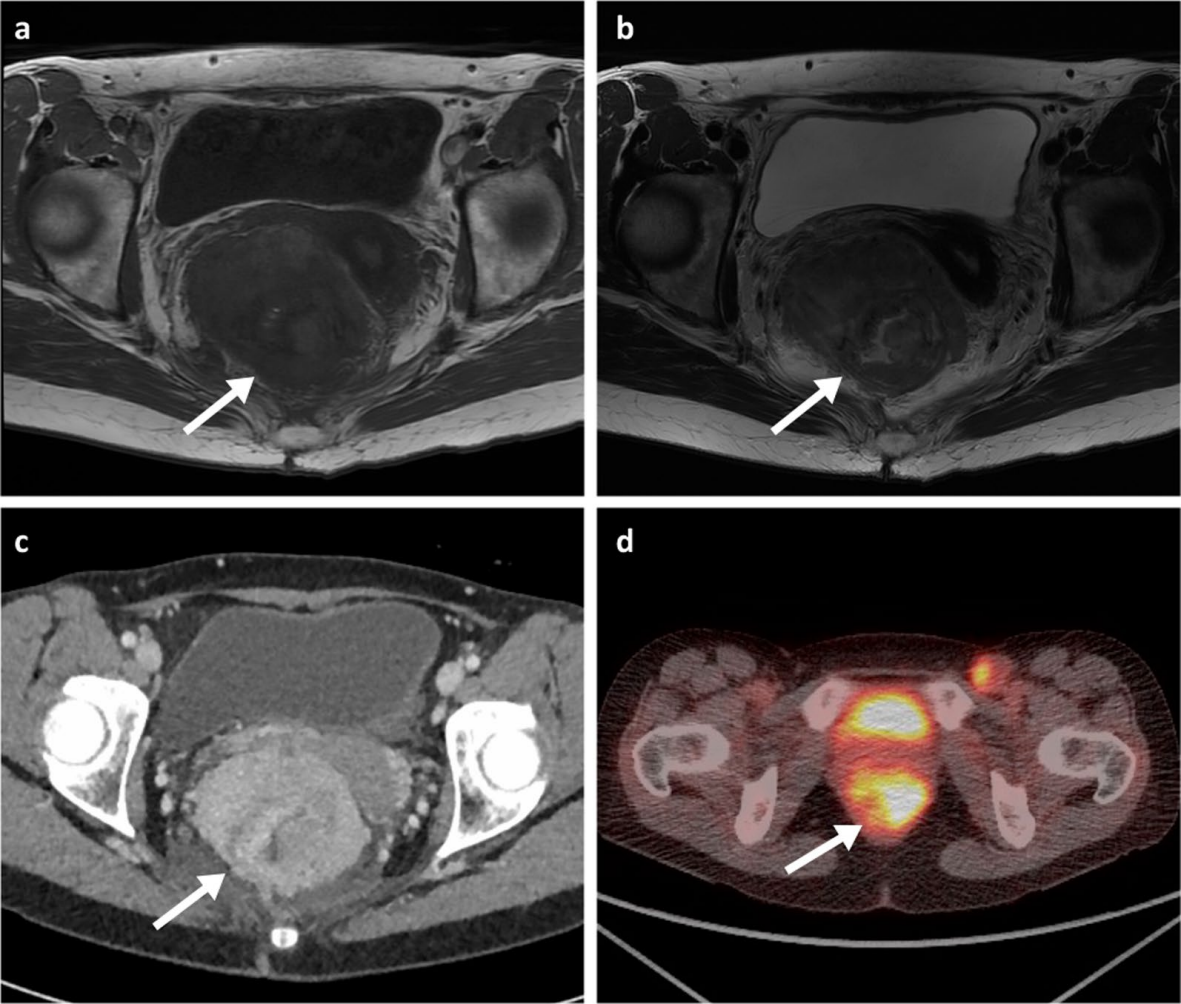
MRI T1WI and T2WI axial (**a**, **b**), portal phase contrast-enhanced CT axial (**c**) and PET-CT axial (obtained two months prior to the shown MRI and CT) (**d**) of a 57-year-old female patient with a large metastatic melanoma anorectal mass (arrows). The mass demonstrates internal foci of higher T1 signal. PET uptake is also seen within a left inguinal node

Clinically, abdominal pain and weight loss are the most common presenting symptoms, with bleeding and a palpable mass being less frequently reported. As with the SB metastases, patients can rarely present with an acute abdomen due to intestinal obstruction, intussusception, perforation, or fistula [27, 38–40]. Though CT imaging may raise initial suspicions on the presence of lesions, colonoscopy has the greatest diagnostic value with high sensitivity and specificity, and also allows collection of tissue for histology. Melanoma metastases in the colon can be multiple and diverse in appearance, spanning from polypoid nodules/masses (Figs. 7b, 9) to ulcerating mural nodules, exo-enteric lesions and infiltrating masses [27]. At the anorectal region, melanoma metastases are usually seen as an intraluminal polypoid or fungating mass in the distal rectum or anal canal (Figs. 10, 11, 12, 13). MRI is the favoured imaging technique for characterisation of lesions at this level due to its improved soft-tissue resolution, with melanoma metastases classically described as having high-signal intensity on T1-weighted (T1W) imaging (Fig. 14) and mixed-signal intensity on T2-weighted (T2W) imaging (Fig. 10) and marked enhancement on post-contrast T1-weighted images [46, 47]. When compared with primary anorectal tumours, MM tends to demonstrate a more perirectal infiltrative growth pattern with a preferential submucosal spread [48] (Fig. 10, 11). Lymphatic involvement and extension to the pelvic side wall and pre-sacral space are common findings at the time of diagnosis (Figs. 11, 12). However, luminal obstruction is rarer in anorectal melanoma than in primary adenocarcinoma, which is frequently obstructive as a consequence of the infiltration and narrowing of the lumen [47].

In suspected resectable disease, metabolic imaging (PET-CT) is often offered to identify other metastatic sites [9], as MM often demonstrates high tracer uptake (Figs. 10, 11). Nevertheless, CT is usually the key technique in the acute setting given its wide availability.

Colonic and anorectal metastases are often associated with late stage disease and consequently poor prognosis. In line with small bowel involvement, surgical resection remains the most common option; not only improving overall survival but also avoiding complications [49, 50].

**Tip 9:** The features of the anorectal metastatic melanoma can sometimes be characteristic on MRI, with high signal on T1WI.

### Peritoneal carcinomatosis

The presence of peritoneal carcinomatosis implies a poor prognosis [51]. Despite being a rare pattern of metastatic melanoma dissemination, peritoneal involvement generally occurs by haematological spread, most commonly in the nodular histological subtype of melanoma [51]. The true incidence and prevalence of peritoneal carcinomatosis from MM are still uncertain, with the few existing studies reporting an estimated prevalence of 0.5% [51].

Peritoneal carcinomatosis can be asymptomatic, but eventually most patients will report symptoms which can vary from uncomfortable to debilitating. Symptoms include abdominal distention, nausea/bloating and intermittent pain due to malignant ascites, or bowel obstruction.

Typically, peritoneal disease is often an incidental finding detected either during staging imaging or during surgery. CT imaging is the preferred method to investigate suspected peritoneal metastases with a reasonable sensitivity for detection (85–93%) [52, 53] and to assess for possible coexisting complications. MR and PET-CT can also detect peritoneal deposits, but offer no significant superiority over CT particularly in the case of small lesions. Nevertheless, the gold standard remains the direct visualisation of the peritoneum through laparoscopy or laparotomy [54].

**Fig. 15:**
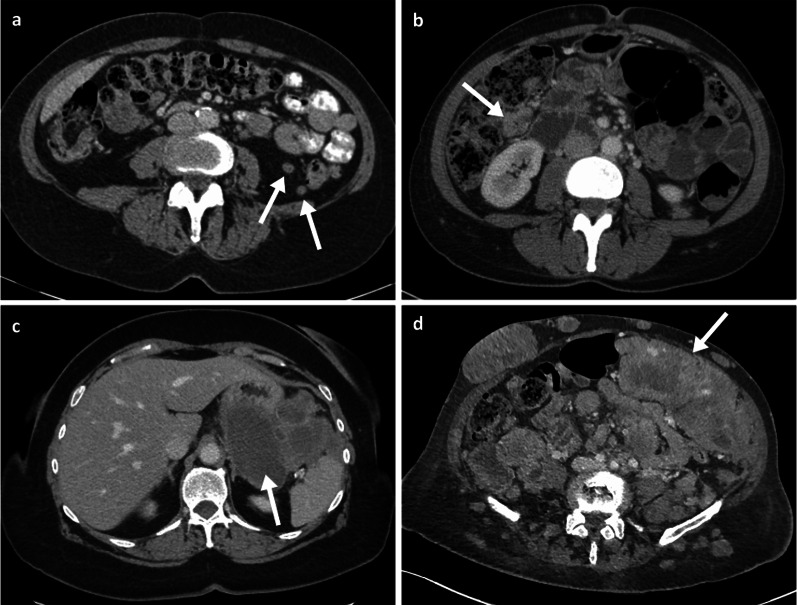
Peritoneal metastatic melanoma cases. **a** Oral and intravenous portal phase contrast-enhanced CT axial of 65-year-old left female with paracolic gutter nodules (arrows). **b** Portal phase contrast-enhanced CT axial of a 45-year-old female patient with a peritoneal deposit adjacent to the hepatic flexure (arrow). **c** Portal phase contrast-enhanced CT axial of a 73-year-old female patient with a large left upper quadrant necrotic peritoneal mass (arrow). **d** Portal phase contrast-enhanced CT axial of a 61-year-old female patient with a large left iliac fossa necrotic peritoneal mass (arrow) as well as numerous peritoneal, retroperitoneal and cutaneous nodules/masses

**Fig. 16:**
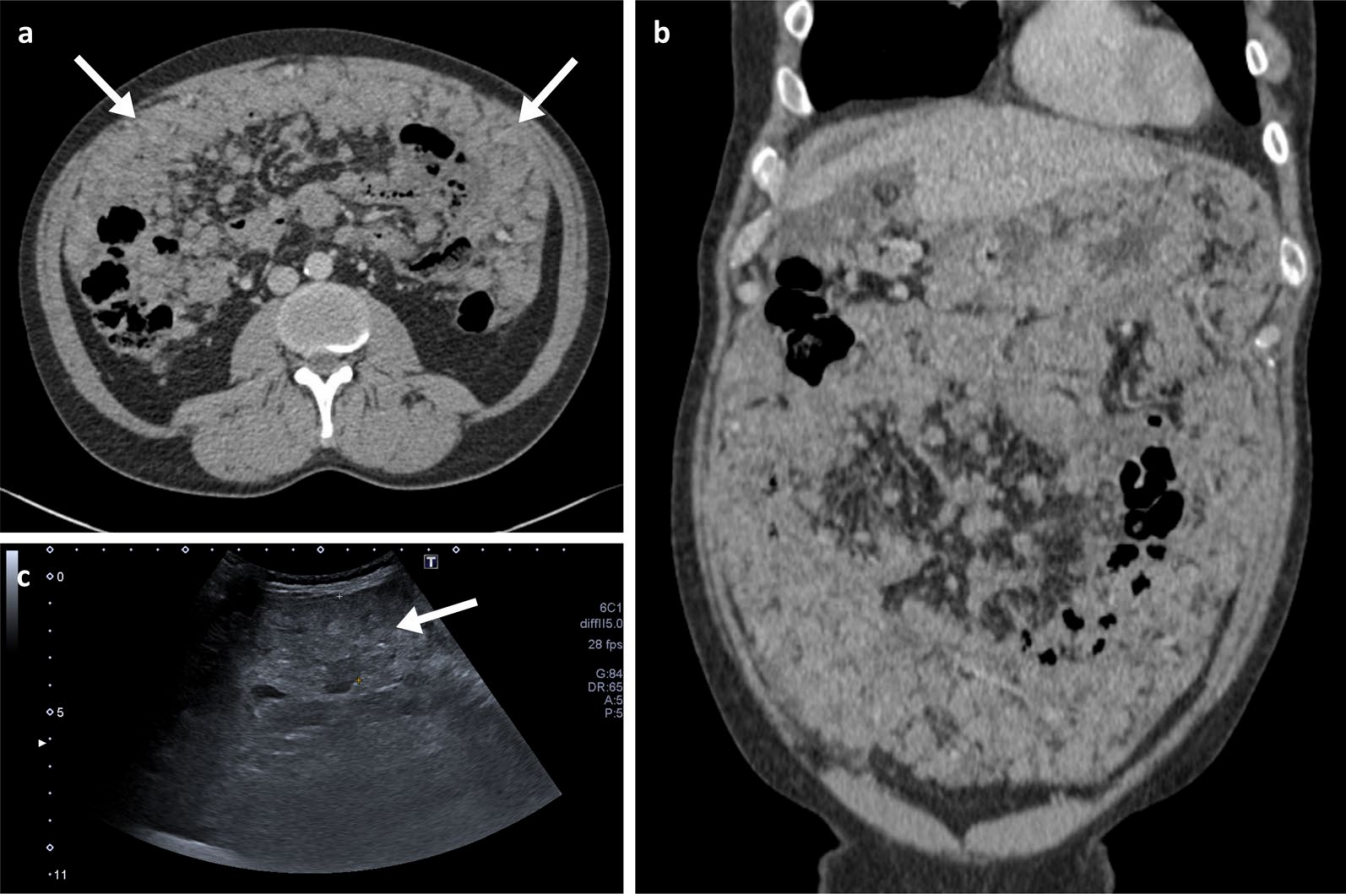
Portal phase contrast-enhanced CT axial (**a**) and coronal (**b**), and abdominal ultrasound (**c**) of a 48-year-old male patient with extensive metastatic melanoma peritoneal disease with innumerable nodules and omental cake (arrows)

Detection of peritoneal disease on imaging requires a trained eye for detection of subtle lesions. Thorough and systematic evaluation of the peritoneal cavity should include careful appreciation of the peritoneal lining, including sites like the lesser sac and splenic hilum (Fig. 15c), mesentery, omentum and serosal covering of the stomach, small and large bowel (Fig. 15b). Peritoneal implants are most often found at the rectouterine pouch, right lower quadrant, sigmoid colon, and right paracolic gutter, due to gravity or arrested of the peritoneal flow [55]. Radiologists should look for specific features like nodular thickening and enhancement of peritoneal reflections, soft-tissue nodules and/or masses (Figs. 6c, 15), stranding and thickening of the omentum (Fig. 14), stranding and distortion of the small bowel mesentery and ascites, especially if loculated [52]. Occasionally, thick stranding of the omentum can be found between the abdominal wall and bowel loops, forming the so-called omental cake (Fig. 16).

Correct identification and reporting of the presence of peritoneal metastases on imaging is of major clinical significance as it will radically impact on patient management in some cases. Unfortunately, peritoneal disease remains an absolute contraindication for surgery with curative intent, but alternative options with modern systemic therapy may benefit these patients.

## Conclusion

Melanoma metastases to the GIT are not uncommon. Oligometastatic lesions can be successfully removed by surgery and offer cure to selected patients, even those whose tumours occur during treatment with modern systemic therapy. CT remains the standard modality for detection, staging and follow-up of these patients. However, detection of GIT metastases can be challenging as they are often subtle, can be multiple and can present with a multitude of morphological appearances.

## Data Availability

Availability of data and materials: The datasets used and/or analysed during the current study are available from the corresponding author on reasonable request.

## Abbreviations

CT: Computer tomography
DWI: Diffusion weighted imaging
GI: Gastrointestinal
GIT: Gastrointestinal tract
MM: Metastatic melanoma
MR: Magnetic resonance
PET: Positron emission tomography
SB: Small bowel

## References

[1] Marks R (2000). Epidemiology of melanoma. Clin Exp Dermatol.

[2] Henley SJ, HMa J, Jemal A (2020). Annual report to the nation on the status of cancer, part I: National Cancer Statistics. Cancer.

[3] Saginala K, Barsouk A, Aluru JS, Rawla P, Barsouk A (2021). Epidemiology of melanoma. Med Sci (Basel).

[4] *SEER*Explorer: An Interactive Website for SEER Cancer Statistics. 7/4/22. https://seer.cancer.gov/explorer.

[5] Ajithkumar T, Parkinson C, Fife K, Corrie P, Jefferies S (2015). Evolving treatment options for melanoma brain metastases. Lancet Oncol.

[6] Schuchter LM, Green R, Fraker D (2000). Primary and metastatic diseases in malignant melanoma of the gastrointestinal tract. Curr Opin Oncol.

[7] Gill SS, Heuman DM, Mihas AA (2001). Small intestinal neoplasms. J Clin Gastroenterol.

[8] Lee MH, Zaheer A, Voltaggio L, Johnson PT, Fishman EK (2019). Clinical time course and CT detection of metastatic disease to the small bowel. Abdom Radiol (NY).

[9] Melanoma: assessment and management. NICE guideline [NG14]. 2022. 31891468

[10] Garbe C, Amaral T, Peris K (2020). European consensus-based interdisciplinary guideline for melanoma—part 1: diagnostics—update 2019. Eur J Cancer.

[11] Ugurel S, Röhmel J, Ascierto PA (2020). Survival of patients with advanced metastatic melanoma: the impact of MAP kinase pathway inhibition and immune checkpoint inhibition—update 2019. Eur J Cancer.

[12] Lens M, Bataille V, Krivokapic Z (2009). Melanoma of the small intestine. Lancet Oncol.

[13] Zoumpos A, Ho A, Loeschhorn-Becker R, Schuppert F (2019). Haemorrhagic small bowel melanoma metastasis: a clinical rarity. BMJ Case Rep.

[14] Melanoma Treatment. 2019, 3 March 2022. https://www.cancer.gov/types/skin/hp/melanoma-treatment-pdq.

[15] Cheung MC, Perez EA, Molina MA (2008). Defining the role of surgery for primary gastrointestinal tract melanoma. J Gastrointest Surg.

[16] Trout AT, Rabinowitz RS, Platt JF, Elsayes KM (2013). Melanoma metastases in the abdomen and pelvis: frequency and patterns of spread. World J Radiol.

[17] Sabanathan S, Eng J, Pradhan GN (1989). Primary malignant melanoma of the esophagus. Am J Gastroenterol.

[18] Spiegelberg H (1895). Ausgebreitete melanosarcomatose als metastase eines tumors der opticusscheide. Arch Pachol Anat.

[19] Dasgupta TK, Brasfield RD (1964). Metastatic melanoma of the gastrointestinal tract. Arch Surg.

[20] Cai JS, Cai HY, Cai JY (2020). Reduced field-of-view diffusion-weighted imaging (DWI) in patients with gastric cancer: comparison with conventional DWI techniques at 3.0T: a preliminary study. Medicine (Baltimore).

[21] Wang J, Yang F, Ao WQ (2019). Primary gastric melanoma: a case report with imaging findings and 5-year follow-up. World J Gastroenterol.

[22] De Vuysere S, Vandecaveye V, De Bruecker Y (2021). Accuracy of whole-body diffusion-weighted MRI (WB-DWI/MRI) in diagnosis, staging and follow-up of gastric cancer, in comparison to CT: a pilot study. BMC Med Imaging.

[23] Mimica M, Tomic I (2002). Endoscopic diagnosis of malignant melanoma metastatic to the stomach. Am J Gastroenterol.

[24] Nelson RS, Lanza F (1978). Malignant melanoma metastatic to the upper gastrointestinal tract: endoscopic and radiologic correlations, form and evolution of lesions, and value of directed biopsy in diagnosis. Gastrointest Endosc.

[25] Booth JB (1965). Malignant melanoma of the stomach. report of a case presenting as an acute perforation and review of the literature. Br J Surg.

[26] Yamamura K, Kondo K, Moritani S (2012). Primary malignant melanoma of the stomach: report of a case. Surg Today.

[27] Othman AE, Eigentler TK, Bier G (2017). Imaging of gastrointestinal melanoma metastases: Correlation with surgery and histopathology of resected specimen. Eur Radiol.

[28] Sinagra E, Sciume C (2020). Ileal Melanoma, a rare cause of small bowel obstruction: report of a case, and short literature review. Curr Radiopharm.

[29] Blecker D, Abraham S, Furth EE, Kochman ML (1999). Melanoma in the gastrointestinal tract. Am J Gastroenterol.

[30] Hadjinicolaou AV, Hadjittofi C, Athanasopoulos PG, Shah R, Ala AA (2016). Primary small bowel melanomas: fact or myth?. Ann Transl Med.

[31] Prakoso E, Selby WS (2007). Capsule endoscopy in patients with malignant melanoma. Am J Gastroenterol.

[32] Kim SY, Kim KW, Kim AY (2006). Bloodborne metastatic tumors to the gastrointestinal tract: CT findings with clinicopathologic correlation. AJR Am J Roentgenol.

[33] Mora JR, Bono MR, Manjunath N (2003). Selective imprinting of gut-homing T cells by Peyer's patch dendritic cells. Nature.

[34] Letsch A, Keilholz U, Schadendorf D (2004). Functional CCR9 expression is associated with small intestinal metastasis. J Invest Dermatol.

[35] Simons M, Ferreira R, Meunier R, Moss S (2016). Primary versus metastatic gastrointestinal melanoma: a rare case and review of current literature. Case Rep Gastrointest Med.

[36] Olatoke SA, Agodirin SO, Adenuga AT (2019). Primary jejunal melanoma as a cause of adult intussusception: a case report and review of literature. Pan Afr Med J.

[37] Bendic A, Durdov MG, Stipic R, Karaman I (2013). Melanoma in the ampulla of Vater. Hepatobiliary Pancreat Dis Int.

[38] Khalid U, Saleem T, Imam AM, Khan MR (2011). Pathogenesis, diagnosis and management of primary melanoma of the colon. World J Surg Oncol.

[39] Miliaras S, Ziogas IA, Mylonas KS, Papadopoulos VN (2018). Primary malignant melanoma of the ascending colon. BMJ Case Rep.

[40] Takahashi-Monroy T, Vergara-Femandez T, Aviles A (2006). Primary melanoma of the colon presenting as ileocecal intussusception. Am J Gastroenterol.

[41] Park J, Ostrowitz MB, Cohen MS, Al-Kasspooles M (2009). A patient with metastatic melanoma of the small bowel. Oncology (Williston Park).

[42] Swetter SM, Carroll LA, Johnson DL, Segall GM (2002). Positron emission tomography is superior to computed tomography for metastatic detection in melanoma patients. Ann Surg Oncol.

[43] Bender GN, Maglinte DD, McLarney JH, Rex D, Kelvin FM (2001). Malignant melanoma: patterns of metastasis to the small bowel, reliability of imaging studies, and clinical relevance. Am J Gastroenterol.

[44] Kim W, Baek JM, Suh YJ, Jeon HM, Kim JA (2004). Ileal malignant melanoma presenting as a mass with aneurysmal dilatation: a case report. J Korean Med Sci.

[45] Mendes Serrao E, Joslin E, McMorran V (2022). The forgotten appearance of metastatic melanoma in the small bowel. Cancer Imaging.

[46] Park HJ, Kim HJ, Park SH (2018). JOURNAL CLUB: primary anorectal melanoma: MRI findings and clinicopathologic correlations. AJR Am J Roentgenol.

[47] Keraliya AR, Krajewski KM, Braschi-Amirfarzan M (2015). Extracutaneous melanomas: a primer for the radiologist. Insights Imaging.

[48] Stefanou A, Nalamati SP (2011). Anorectal melanoma. Clin Colon Rectal Surg.

[49] Reddy P, Walker C, Afonso B (2014). A rare case of metastatic malignant melanoma to the colon from an unknown primary. Case Rep Gastrointest Med.

[50] Wysocki WM, Komorowski AL, Darasz Z (2004). Gastrointestinal metastases from malignant melanoma: report of a case. Surg Today.

[51] Flanagan M, Solon J, Chang KH (2018). Peritoneal metastases from extra-abdominal cancer—a population-based study. Eur J Surg Oncol.

[52] Levy AD, Shaw JC, Sobin LH (2009). Secondary tumors and tumorlike lesions of the peritoneal cavity: imaging features with pathologic correlation. Radiographics.

[53] Halvorsen RA, PanushkaOakleyLetourneau CGJJG, Adcock LL (1991). Intraperitoneal contrast material improves the CT detection of peritoneal metastases. AJR Am J Roentgenol.

[54] Coccolini F, Gheza F, Lotti M (2013). Peritoneal carcinomatosis. World J Gastroenterol.

[55] Nougaret S, Addley HC, Colombo PE (2012). Ovarian carcinomatosis: how the radiologist can help plan the surgical approach. Radiographics.

